# Data for iTRAQ profiling of micro-vesicular plasma specimens: In search of potential prognostic circulatory biomarkers for Lacunar infarction

**DOI:** 10.1016/j.dib.2015.07.021

**Published:** 2015-07-26

**Authors:** Arnab Datta, Siu Kwan Sze

**Affiliations:** School of Biological Sciences, Nanyang Technological University, Singapore, Singapore

## Abstract

To discover potential prognostic biomarkers of Lacunar infarction (LACI), here we present quantitative proteomics data of plasma microvesicle-enriched fraction derived by comparative isobaric profiling of three groups of prospectively followed-up LACI patients (LACI – no adverse outcome, LACI –recurrent vascular event and LACI – cognitive decline) and a demographically matched control group. We confidently (unused prot score >3, FDR=1.1%) identified 183 proteins, 43 out of which were significantly regulated (*p*-value<0.05) in at least one of the three LACI groups in comparison to control group. Bioinformatics analysis and data mining revealed upregulation of brain-specific proteins including myelin basic protein, proteins of coagulation cascade (e.g., fibrinogen alpha chain, fibrinogen beta chain) and focal adhesion (e.g., integrin alpha-IIb, talin-1, and filamin-A) while albumin was downregulated in both groups of patients with adverse outcome. The data of this study are also in line with our previously published article entitled “Discovery of prognostic biomarker candidates of Lacunar infarction by quantitative proteomics of microvesicles enriched plasma” by Datta et al. (2014). The raw data had been deposited to the ProteomeXchange consortium with identifier PXD000748.

## Specifications table

**Table:** 

Subject area	*Biotechnology*, *medicine*
More specific subject area	*Quantitative proteomics*, *Lacunar infarction*, *Prognostic biomarker*
Type of data	*Table*, *figure and excel file*
How data was acquired	*QStar Elite mass spectrometer*(*Applied Biosystems*/*MDSSciex*, Foster City, CA, USA) *coupled with online microflow HPLC system*
Data format	Raw:*.wiff files generated* with Analyst QS2.0 software (Applied Biosystems).Analyzed: *Proteinpilot Group files processed by ProteinPilot™ software*3.0 (*revision number* 114732; *Applied Biosystems*)
Experimental factors	*Micro-vesicles were isolated from plasma specimens by sequential normal and ultra-centrifugation. They were lyophilized and dissolved in sodium dodecyl sulfate containing dissolution buffer prior to in-gel digestion*, *isoberic labeling*, *chromatographic fractionation and mass spectrometry*.
Experimental features	• *Multi-dimensional liquid chromatography coupled with mass spectrometry* ✓ *4-plex Isobaric tags for relative and absolute quantification* (*iTRAQ*)( *Applied Biosystems*) ✓ *Electrostatic repulsion and hydrophilic interaction chromatography (ERLIC)*
Data source location	*Singapore*
Data accessibility	*All data are available with this article. The proteomics raw data files can be found at ProteomeXchange with identifier PXD000748.*

## Value of the data

**Table:** 

• Our study is the first of its kind where an iTRAQ-guided discovery proteomics approach was used to identify potential prognostic circulatory biomarkers of ischemic stroke. • Bioinformatics analysis and data mining revealed upregulation of brain-specific proteins including myelin basic protein, proteins of coagulation cascade (e.g., fibrinogen alpha chain, fibrinogen beta chain) and focal adhesion (e.g., integrin alpha-IIb, talin-1, and filamin-A) while albumin was downregulated in both groups of LACI patients with adverse outcome. • The short-listed candidate prognostic biomarkers from this data set may stimulate validation studies on independent cohort of individual LACI patients. • In absence of clinically proven circulatory biomarkers, these candidates can also be tried as surrogate markers in LACI-related clinical trials to check the consequences of therapeutic interventions. • From an academic standpoint, this data set may offer important insight into the mechanisms of poor prognosis following LACI.

## 1 Experimental design, materials and methods

Biomarker for prognosis of ischemic stroke is a relatively new concept compared to biomarkers for diagnosis. No single or panel of blood-based biomarkers has been validated by clinical trials for stroke or related secondary complications. The short-term prognosis of ischemic cerebral small-vessel disease (SVD), including Lacunar infarction (LACI) is more favorable, with almost negligible early mortality, absence of neuropsychological impairment and an excellent neurological recovery. However, LACI causes an increase in the mid- or long-term risk of recurrent vascular disorder and of different types of vascular dementia or neuropsychological abnormalities [2]. On the other hand, routinely used brain imaging techniques are often not sufficient to produce high resolution images for patients with LACI affecting small arterioles (0.2–0.8 mm) and having smaller size than the larger territorial infarct seen in non-lacunar strokes [3]. Hence, prognostic biomarkers for LACI will complement the existing symptomatic and diagnostic protocols used for patient management.

Plasma microvesicle is a good source of disease biomarkers that entered the circulatory system following their release by cells from various tissues including the cells of central nervous system [4], [5], [6], [7]. Ischemic SVD is well-known to cause an endothelial dysfunction and a diffuse increase in the blood brain barrier (BBB) permeability that may facilitate the leakage of microvesicles in the general circulation [8]. Here, we hypothesize that the brain cells of LACI patients with poor prognosis under the influence of ischemic stress may release microvesicles into circulation through the compromised BBB during its evolution. Accordingly, we targeted plasma microvesicle enriched fractions for profiling through iTRAQ‐2D‐LC‐MS/MS strategy [9], [10], [11], [12]. An offline weak anion exchange (WAX) chromatography (PolyLC, Columbia, MD, USA) was followed by an online reverse phase chromatography and tandem mass spectrometry with QSTAR Elite Hybrid MS (Applied Biosystems/MDS-SCIEX, Foster City, CA, USA). The mass spectrometric data analysis was performed using Analyst QS 2.0 (Applied Biosystems) and ProteinPilot Software (v 3.0, Revision Number: 114 732, Applied Biosystems) software while the bioinformatics analysis was performed with DAVID and GenePattern (version 3.3.3) software.

## 2 Collection of samples and clinical information

All patients with recent transient ischemic attacks (TIA) or nondisabling ischemic stroke who were seen at the Singapore General Hospital between 1999 and 2005 were screened for eligibility for the European Australasian Stroke Prevention in Reversible Ischemia Trial (ESPRIT). The ESPRIT trial was registered under http://clinicaltrials.gov with the identifier NCT00161070. Patients recruited into ESPRIT were eligible to enter a cognitive substudy (ESPRIT-Cog). Detailed methodology of ESPRIT and ESPRIT-cog including the exclusion criteria have been reported previously [1], [13], [14]. Stroke subtype was classified according to the Oxfordshire Community Stroke Project as total anterior circulation infarct, partial anterior circulation infarct, posterior circulation infarct, or LACI [15]. The patients were eligible if they were within six-months of a TIA (including transient monocular blindness) or nondisabling ischemic stroke [grade ≤3 on the modified Rankin scale (mRS)] of presumed arterial origin [16].The control plasma was collected from non-stroke subjects at the same site during 2004–2006.

Risk factor information (e.g. diabetes mellitus status, hypertension, hyperlipidemia, smoking status) was collected at baseline. The cognitive status of the patients was determined by trained research psychologists using standard neuropsychological test battery that has been validated for use in Singapore. Details of the procedure have been described previously [1], [14], [17]. Diagnoses of dementia were made according to the DSM-IV criteria [18]. Diagnoses of various stages of dementia were made after each patient׳s baseline and follow up visits.

## 3 Experimental design guided by outcome measures

Of the 458 patients enrolled in the Singapore General Hospital׳s site of the ESPRIT trial, 26 (6%) refused to provide blood samples and were excluded from the study. Hence, 432 consented to participate in the ESPRIT-Cog substudy. Of these 432 patients, 275 (64%) had LACI, which was the population of interest for the current study. Further, 10 were excluded as they had dementia at baseline. Several patients had insufficient plasma samples or dropped out during the follow up period and were therefore excluded. Representative 45 (45/265, 17%) LACI patients were selected for this discovery proteomics study. Notably, there were no differences in demographic characteristics between these 45 and remaining 220 LACI patients of this cohort (data not shown).

**Fig. 1:**
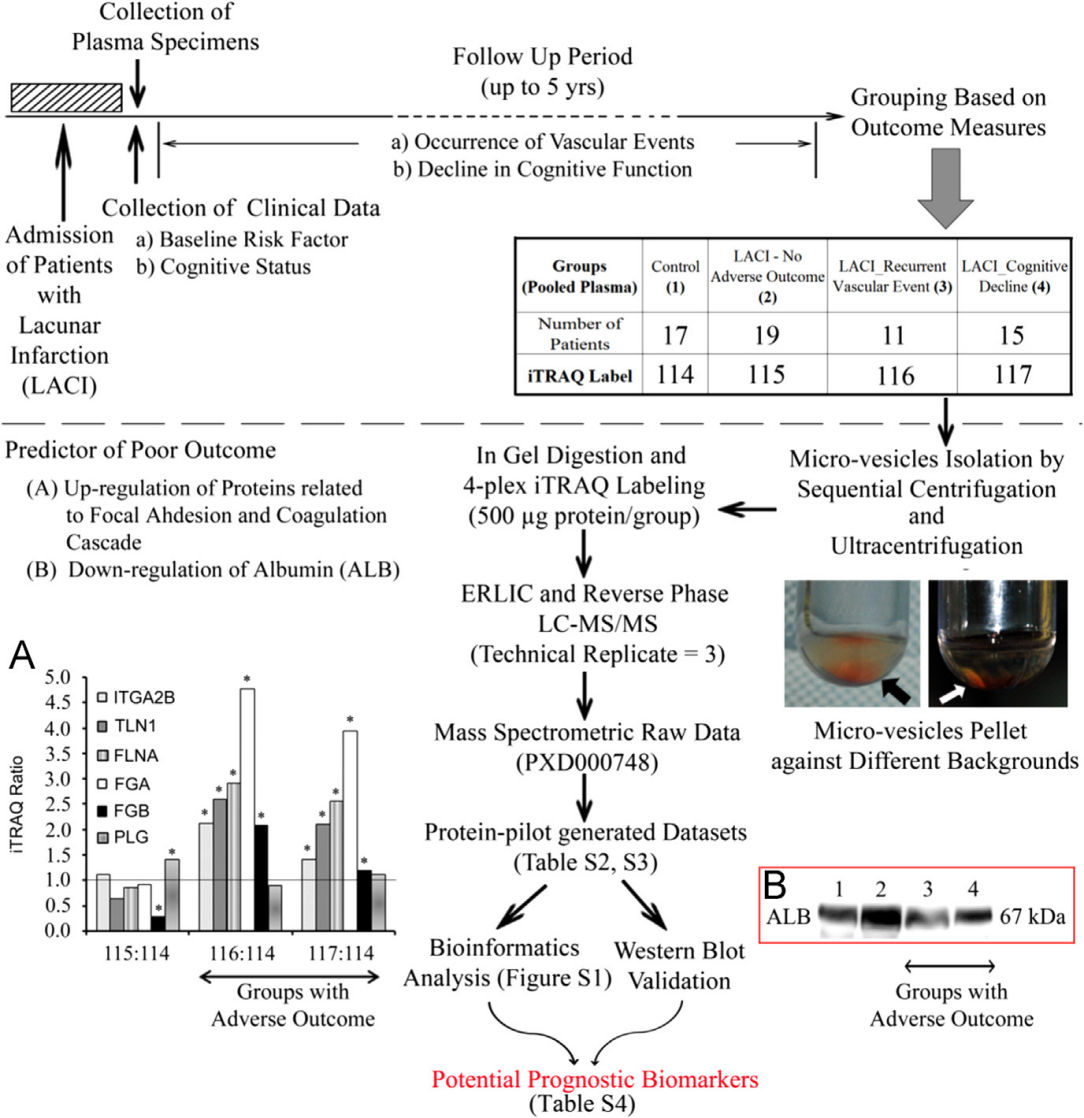
Schematic representation of the experimental design along with key findings and supporting data.

The LACI patients were followed up annually for up to 5 years (median follow up 3 years; interquartile range, 2 years) to monitor for the occurrence of any vascular event or for change in the cognitive status. Strokes, peripheral artery disease, intracranial bleeds, and any cardiac ischemia (stable and unstable angina, myocardial infarctions) or deaths from any of the above were considered to be a recurrent vascular event. Any LACI patient having a recurrence of vascular event during the follow-up period was included in the group called “recurrent vascular event” [14], [19]. The patients whose cognitive status declined from the respective baseline status during the course of the prospective study had been assigned to the “cognitive decline” group. Patients who did not suffer a recurrent vascular event or cognitive decline during this period were grouped as “LACI, no adverse outcome”. Accordingly, plasma samples of 45 LACI patients were divided into three groups based on the outcome variables (LACI – no adverse outcome, *n*=19; LACI – recurrent vascular events, *n*=11; LACI – cognitive decline but no recurrent vascular events, *n*=15). The age-matched control group had 17 subjects who never had a stroke or cancer and were cognitively normal at the baseline. The experimental design is depicted in Fig. 1.

The demographic characteristics, baseline risk factors and cognitive classifications of the study population stratified by outcome measures and control group are summarized in Table S1. The average age of the recruited subjects was 61±10 years; 55% were males and 92% were Chinese. No significant difference was observed between three groups of LACI patients in terms of most of the baseline risk factors except ‘smoking’ (*H*(2)=7.276, *p*=0.026).

The plasma samples were pooled group-wise before processing. A microvesicle-enriched fraction was isolated by sequential centrifugation combined with ultracentrifugation and labeled with isobaric tags that was followed by 2D LC-MS/MS analysis to improve the depth of identification and quantification. The iTRAQ samples were injected thrice in the LC-MS/MS analysis (technical replicate=3).

## 4 Pre-proteomics sample preparation

### 4.1 Separation of microvesicle-enriched fraction by sequential centrifugation

Frozen individual plasma samples were thawed on ice and pooled in a group-wise manner to obtain four tubes containing around 5 ml of plasma specimens from each group. The samples were subjected to sequential centrifugation to enrich the microvesicles using a modified protocol as described previously [20], [21]. Briefly, sonicated plasma (5X 1 min) was centrifuged at 4000*g* twice for 30 min and then at 12,000*g* for 30 min to collect and remove the pellets. The resulting supernatant was subsequently diluted approx. five times with ice-cold 1X PBS before doing ultra-centrifugation at 30,000*g* for 2 h to collect the pellet of plasma membrane derived vesicles or microparticles for a separate study. The supernatant was ultra-centrifuged again at 200,000*g* for 2 h 15 min to collect the microvesicle pellet (Fig. 1). The microvesicle pellets were washed at least twice with 1X PBS and were lyophilized. The lyophilisate was dissolved using 50–100 µl of ice-cold dissolution buffer [6% sodium dodecyl sulfate; 20 mM dithiothreitol, 100 mM Tris–HCl with Complete Protease Inhibitor Cocktail (COMPLETE, (Roche; Mannheim, Germany)), pH 7.75] by brief vortexing. Protein quantization was performed using 2-D Quant kit (Amersham Biosciences, Piscataway, NJ, USA).

## 5 Proteomics

### 5.1 In-gel tryptic digestion and isobaric labeling

The samples (500μg/condition) were subjected to denaturing PAGE using a 4–6–25% gel following an identical procedure as described previously [10], [11]. Briefly, the diced gel bands were extensively washed with 25 mM triethylammonium bicarbonate (TEAB) in 50% acetonitrile (ACN) to completely remove Tris HCl and detergent before reduction (in 5 mM tris (2-carboxyethyl) phosphine, 25 mM TEAB, 60 °C, 1 h) and alkylation (in 10 mM methyl methanethiosulfonate in 25 mM TEAB in dark, RT, 45 min). They were digested overnight (12.5 ng/μl of sequencing-grade modified trypsin) ((Promega, Madison, WI, USA), in 50 mM TEAB, 2% ACN) at 37 °C. Subsequently, the peptides were extracted and dried before reconstituting them into 0.5 M TEAB and labeled with respective isobaric tags of 4-plex iTRAQ Reagent Multi-Plex kit (Applied Biosystems) for 2 h as follows: Control, 114; LACI – no adverse outcome, 115; LACI – recurrent vascular events, 116; LACI – cognitive decline but no recurrent vascular events, 117 (Fig. 1). The labeling reaction was stopped by addition of water in each tube before combining all four groups for vacuum centrifugation.

## 6 Electrostatic repulsion and hydrophilic interaction chromatography (ERLIC)

The dried iTRAQ-labeled peptides were desalted by Sep-Pak C18 SPE cartridges (Waters, Milford, MA, USA). A modified ERLIC with volatile salt-containing buffers was adopted using a hydrophilic WAX column (PolyWAX LP, 200×4.6 mm^2^; 5 µm; 300 Å) (PolyLC) that was conditioned overnight by periodic and intermittent washing with the chromatographic buffers and 0.5 M KCl [11], [22]. The iTRAQ-labeled peptides were reconstituted in 200 µl of Buffer A (10 mM NH_4_HCO_2_, 85% ACN, 0.1% formic acid (FA)) and fractionated on a Prominence HPLC system (Shimadzu, Kyoto, Japan) in a 65 min gradient with Buffer B (30% ACN, 0.1% FA). The HPLC gradient was composed of 100% buffer A for 10 min; 0–25% buffer B for 35 min; then 25–100% buffer B for 10 min; followed by 100% buffer B for 10 min. The chromatogram was recorded at 280 nm. Eluted fractions were collected in every 1 min, and then pooled into 34 fractions depending on the peak intensities, before drying them in a vacuum centrifuge. They were stored at −20 °C till MS analysis.

## 7 Reverse phase LC-MS/MS analysis using QSTAR

The iTRAQ-labeled peptides were reconstituted with 0.1% FA, 3% ACN and analyzed using a HPLC system (Shimadzu) coupled with QSTAR Elite Hybrid MS (Applied Biosystems/MDS-SCIEX) as described previously with minor modifications. Briefly, most of the LC parameters for a 90 min gradient including column configuration, gradient and flow rate were kept constant except the mobile phase A composition (0.1% FA in 3% ACN) and sample injection volume (15 µl/injection). Regarding MS parameters, the precursors with a mass range of 300–1600*m*/*z* and calculated charge of +2 to +5 were selected for the fragmentation. The selected precursor ion was dynamically excluded for 20 s with a 50 mDa mass tolerance. The maximum accumulation time was set at 1.0 s. All other MS parameters were kept identical as reported previously [10].

## 8 Mass spectrometric raw data analysis

The Analyst QS 2.0 software (Applied Biosystems) was used for the spectral data acquisition. Spectra acquired from each of the technical replicates were submitted alone and together to ProteinPilot Software (Applied Biosystems) for peak list generation, protein identification and quantification against the concatenated target-decoy Uniport human database (191242 sequences, downloaded on 12 March 2012 from www.uniprot.org). The false discovery rate (FDR) of peptide identification was set to be less than 1% (FDR=2.0×decoy_hits/total_hits). Details of the analysis strategy have been described previously [10]. The proteins and peptides that are identified and quantified by iTRAQ experiment were exported from ProteinPilot and listed in the Table S2 (Protein summary) and Table S3 (Peptide summary). Hundred eighty three proteins were identified when a strict cut-off of unused prot score >3 (corresponds to 99.9% confidence) was adopted to keep the FDR at 1.1%. However, 288, 377 and 458 proteins were identified with unused score ≥2 (>99% confidence), >1.3 (>95% confidence) and >1.0 (>90% confidence) respectively. Filtering the protein list with a *p*-value cut-off of <0.05 resulted in a shortlist containing 17, 33 and 28 proteins for the three ratios (i.e. 115/114, 116/114 and 117/114) respectively after excluding the keratins from the list. Overall, 43 proteins having at least one ratio with an acceptable level of confidence were shortlisted for the bioinformatics analysis to retrieve useful biological trends (Table S4).

## 9 Bioinformatics analysis

The open-source software DAVID (http://david.abcc.ncifcrf.gov/) and GenePattern (www.broadinstitute.org/cancer/software/genepattern/) were used for the enrichment and clustering analysis by submitting Uniprot accession numbers of the short-listed 43 proteins [23], [24]. Different attributes of DAVID such as gene ontology (GO), pathway, protein interaction, keywords and tissue specificity were used to extract out hidden trends and enrichment of certain groups of proteins. DAVID uses modular enrichment analysis where the term–term/gene–gene relationships are considered for enrichment *p-*value calculation. It calculates the probability of the number of genes in the list that hit a given biology class as compared to pure random chance with the aid of Fisher׳s exact test. To check the enrichment, *p*-value≤0.01 and FDR <1% were used as a cut-off. Searching for enriched pathways using various modules (e.g. KEGG, Biocarta, Reactome) showed ‘complement and coagulation cascades’, ‘intrinsic prothrombin activation pathway’ or ‘integrin cell surface interactions’ (e.g. ITGA2B, TLN1, FGB and FGA) as significantly over-represented (Fig. 1A, Table S4) with a differential trend among the groups of ‘no adverse’ and ‘adverse’ outcome.

Hierarchical clustering algorithm of GenePattern uses Pearson correlation analysis between Log_2_-transformed ratios of each protein with various conditions to generate a tree structure, which is referred to as dendrogram. The clustering analysis result classified the proteins into two major clusters (Figure S1I and II) separating the up- and downregulated proteins in adverse outcome groups.

## 10 Western Blot (WB) Analyses

ALB was one of the most deregulated candidates as per the iTRAQ data in the groups with adverse outcome (Table S4, Figure S1). WB was performed after SDS-PAGE by probing with anti-ALB primary antibody (albumin, 1:5000, rabbit polyclonal; Abcam, Cambridge, UK) to check the technical reliability of the iTRAQ result. 20 µg proteins were used for WB. Immunoreactivity was detected using an HRP chemiluminescent substrate reagent kit (Invitrogen, Carlsbad, CA, USA). The WB result showed consistent trends with the iTRAQ result (Fig. 1B).

## 11 Statistical Analyses

All statistical analyses were performed using SPSS 13.0 for Windows software (SPSS Inc.). One-way ANOVA followed by post-hoc Tukey test was used for scale variables such as age. Nonparametric Kruskal–Wallis *H* Test was used for comparing ordinal variables such as demographic characteristics and baseline risk factors. Statistical significance was accepted at *p*<0.05.

## 12 Limitations

Our study has few limitations. First, the LACI patients were recruited over a longer span (6 years. vs 2 years.) compared to the control subjects, making the average storage duration longer for LACI patients than the control subjects. Second, the samples had been stored for at least 5 years (up to maximum 12 years) at −80 °C, which should be taken into account before comparing the data with similar studies during a meta-analysis. Notably, major part of the waiting time is included in the study duration as long-term outcome variables were targeted to discover potential prognostic biomarkers. Further, similar storage time is a common occurrence in biomarker studies and shown to adequately preserve the quality of frozen samples when compared with freshly collected specimens for various circulatory proteins such as insulin-like growth factor-I and transforming growth factor *β* [25], [26].

## Conflict of interest

A part of the work has been used to file a US provisional patent (Patent Filing number: 61/876361) on “Plasma Microvesicle Biomarker for Diagnosis and Prognosis of Stroke.” The inventors are Sze Siu Kwan, Arnab Datta and Xavier Gallart-Palau from Nanyang Technological University and Christopher Chen from National University of Singapore. The provisional patent is granted.
