# Supplementary material for: Data for iTRAQ profiling of micro-vesicular plasma specimens: In search of potential prognostic circulatory biomarkers for Lacunar infarction
Source: Data Brief. 2015 Jul 26;4:510–7. doi: 10.1016/j.dib.2015.07.021 (PMC4783520; doi:10.1016/j.dib.2015.07.021)
Supplement: Supplementary file 1 — Supplementary Material [file mmc1.doc]

Figure S1.


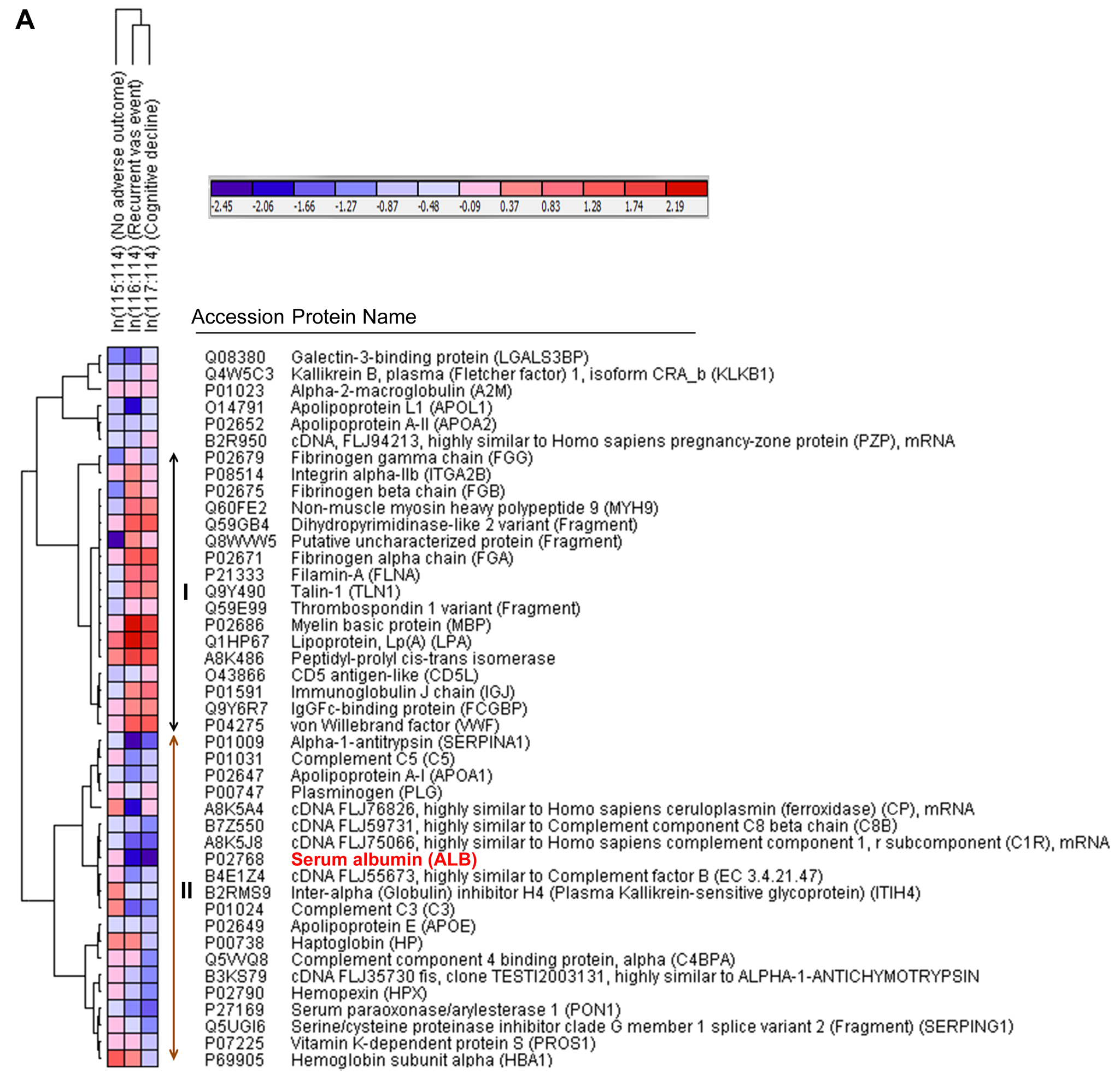


Figure Legend

**Hierarchical clustering of the filtered list of 43 proteins from microvesicle-enriched plasma**. Log2-transformed ratios (e.g. ln (115/114)) of each protein (row) were presented for all conditions (column). Pearson correlation was applied for the measurement of row and column distance. Globally normalized view was presented here. The color scale of the heat map ranges from saturated blue (value, - 2.45) to saturated red (value, 2.19) in the natural logarithmic scale. The proteins were mainly clustered into two parts as shown by I (up-regulated in adverse outcome groups) and II (down-regulated in the adverse outcome groups). The pattern of regulation was similar between recurrent vascular event and cognitive decline group amid subtle differences in magnitudes. MBP and ALB were the two most regulated proteins. The protein names and accession numbers were taken from the Uniprot protein database. The gene symbols are provided within brackets along with the protein name, wherever available. ALB (marked red) was validated with western blot analysis using pooled plasma samples.
