# Supplementary material for: Data for iTRAQ profiling of micro-vesicular plasma specimens: In search of potential prognostic circulatory biomarkers for Lacunar infarction
Source: Data Brief. 2015 Jul 26;4:510–7. doi: 10.1016/j.dib.2015.07.021 (PMC4783520; doi:10.1016/j.dib.2015.07.021)
Supplement: Supplementary file 2 — Supplementary Material [file mmc2.doc]

**Table S**1. Demographic Characteristics of the Patient Population Stratified by the Outcome Measures

| Characteristic N (%) | No adverse outcome (N = 19) | Recurrent vascular events (stroke + MI) (N = 11) | Cognitive decline (no recurrent vascular events) (N = 15) | Healthy control (N = 17) |
| --- | --- | --- | --- | --- |
| Age, Mean (SD) ^†^ | 61 (9) | 65 (10) | 66 (9) | 56 (9) |
| Sex, Male | 17 (90) | 8 (73) | 5 (33) | 4 (26) |
| Ethnicity, Chinese | 17 (90) | 8 (73) | 15 (100) | 17 (100) |
| Diabetes mellitus | 7 (37) | 2 (18) | 7 (47) | 6 (35) |
| Hypertension | 11 (58) | 9 (82) | 12 (80) | 10 (59) |
| Previous stroke | 0 (0) | 3 (27) | 4 (27) | None |
| Hyperlipidemia | 8 (42) | 4 (36) | 9 (60) | 10 (59) |
| Ever smoker | 5 (26) | 6 (55) | 1 (7) | 1 (6) |
| Previous ischemic heart disease | 2 (11) | 2 (18) | 1 (7) | 3 (18) |
| Previous myocardial infarction | 0 (0) | 0 (0) | 1 (7) |
| Previous angina | 2 (11) | 2 (18) | 0 (0) |
| Previous peripheral artery disease | 0 (0) | 0 (0) | 0 (0) | None |
| Baseline cognitive classification |  |  |  |  |
| NCI | 13 (68) | 2 (18) | 8 (53) | None |
| CIND-mild | 4 (21) | 6 (55) | 6 (40) |
| CIND-moderate | 3 (16) | 3 (27) | 1 (7) |

All values are reported as: N(%), where N indicates the number of observations. ^†^Values are expressed as: Mean (±standard deviation). NCI, no cognitive impairment; CIND, cognitive impairment no dementia.
