# Supplementary material for: Data for iTRAQ profiling of micro-vesicular plasma specimens: In search of potential prognostic circulatory biomarkers for Lacunar infarction
Source: Data Brief. 2015 Jul 26;4:510–7. doi: 10.1016/j.dib.2015.07.021 (PMC4783520; doi:10.1016/j.dib.2015.07.021)
Supplement: Supplementary file 5 — Supplementary Material [file mmc5.doc]

**Table S4. The List of Qualified and Regulated Proteome from Microvesicle-enriched Plasma *^a^***

| N | Unused | %Cov (95) | Accession Number | Protein name | Gene Symbol | Peptides (95%) | 115:114 | 116:114 | 117:114 | GO/pathway |
| --- | --- | --- | --- | --- | --- | --- | --- | --- | --- | --- |
| 1 | 1099.8 | 86.2 | P01023 | Alpha-2-macroglobulin | A2M | 2474 | 1.00 | **1.02** | **1.07** | CCC, EIA, RTW, ECS |
| 3 | 257.0 | 68.8 | P01024 | Complement C3 | C3 | 291 | **1.54** | **0.22** | **0.30** | CCC, EIA, RTW, ECS, IIR |
| 4 | 226.6 | 83.7 | P02768 | Serum albumin***** | ALB | 433 | 1.16 | **0.14** | **0.12** | ECS |
| 7 | 155.1 | 47.9 | P02671 | Fibrinogen alpha chain | FGA | 267 | 0.92 | **4.79** | **3.94** | CCC, RTW, ECS |
| 10 | 124.2 | 78.0 | P02675 | Fibrinogen beta chain | FGB | 209 | **0.29** | **2.09** | **1.20** | CCC, ECS |
| 11 | 106.0 | 67.0 | P00738 | Haptoglobin | HP | 171 | 1.94 | 1.80 | **0.58** | ECS |
| 13 | 99.4 | 57.2 | P02679 | Fibrinogen gamma chain | FGG | 193 | **0.30** | **1.38** | **0.55** | CCC, RTW, ECS |
| 18 | 71.1 | 15.7 | P04275 | von Willebrand factor | VWF | 44 | **1.36** | **3.94** | **4.25** | FA, CCC, RTW, ECS |
| 22 | 52.9 | 18.1 | P01031 | Complement C5 | C5 | 29 | **1.32** | **0.36** | 0.56 | CCC, EIA, RTW, ECS, IIR |
| 23 | 51.9 | 8.8 | Q9Y6R7 | IgGFc-binding protein | FCGBP | 31 | 1.02 | **1.57** | **1.84** | ECS |
| 25 | 49.1 | 41.7 | *Q5VVQ8* | Complement component 4 binding protein, alpha | C4BPA | 40 | 1.27 | **1.42** | **0.35** | CCC, RTW, ECS, IIR |
| 27 | 45.3 | 68.9 | P02647 | Apolipoprotein A-I | APOA1 | 30 | 0.77 | **0.33** | **0.52** | LT, ECS |
| 30 | 41.9 | 34.5 | Q08380 | Galectin-3-binding protein***** | LGALS3BP | 44 | **0.30** | **0.26** | 0.78 | ECS |
| 31 | 41.0 | 14.0 | *Q60FE2* | Non-muscle myosin heavy polypeptide 9 | MYH9 | 25 | 0.49 | **3.19** | **2.05** |  |
| 33 | 39.8 | 11.1 | P21333 | Filamin-A | FLNA | 21 | 0.88 | **2.94** | **2.58** | FA, ECS |
| 34 | 38.6 | 11.0 | Q9Y490 | Talin-1 | TLN1 | 19 | 0.65 | **2.61** | **2.11** | FA, ECS |
| 36 | 37.4 | 69.7 | P69905 | Hemoglobin subunit alpha | HBA1 | 56 | **3.87** | **1.89** | 0.49 |  |
| 37 | 35.2 | 47.3 | O43866 | CD5 antigen-like | CD5L | 28 | **0.46** | 0.86 | 1.04 | ECS |
| 40 | 32.6 | 38.5 | P01009 | Alpha-1-antitrypsin | SERPINA1 | 21 | 0.75 | **0.09** | **0.20** | CCC, EIA, RTW, ECS |
| 42 | 30.3 | 22.3 | P00747 | Plasminogen | PLG | 14 | **1.43** | 0.91 | 1.14 | CCC, RTW, ECS |
| 45 | 28.5 | 23.8 | *Q4W5C3* | Kallikrein B, plasma (Fletcher factor) 1, isoform CRA_b | KLKB1 | 17 | **0.59** | **0.55** | 1.02 | CCC, RTW, ECS |
| 48 | 24.9 | 20.3 | P07225 | Vitamin K-dependent protein S | PROS1 | 13 | 1.43 | 1.05 | **0.49** | CCC, EIA, RTW, ECS |
| 50 | 24.0 | 15.0 | *B2RMS9* | Inter-alpha (Globulin) inhibitor H4 (Plasma Kallikrein-sensitive glycoprotein) | ITIH4 | 14 | **1.69** | 0.73 | 0.75 | EIA, RTW, ECS |
| 51 | 21.4 | 29.6 | O14791 | Apolipoprotein L1 | APOL1 | 15 | 0.61 | **0.14** | 0.69 | LT, ECS, IIR |
| 52 | 21.3 | 14.7 | *Q1HP67* | Lipoprotein, Lp(A) | LPA | 14 | **2.81** | **9.12** | **7.38** | LT, EIA, RTW, ECS |
| 53 | 19.9 | 27.9 | P02790 | Hemopexin | HPX | 12 | 1.18 | 0.51 | **0.30** | ECS |
| 61 | 16.0 | 27.8 | P02649 | Apolipoprotein E | APOE | 8 | 0.69 | **0.65** | **0.49** | LT, ECS |
| 67 | 13.7 | 8.0 | P08514 | Integrin alpha-IIb***** | ITGA2B | 6 | 1.12 | **2.13** | 1.42 | FA |
| 74 | 12.5 | 36.5 | P01591 | Immunoglobulin J chain | IGJ | 16 | 0.77 | **1.96** | **2.75** | ECS |
| 76 | 12.2 | 20.6 | P27169 | Serum paraoxonase/arylesterase 1 | PON1 | 6 | 0.75 | **0.41** | **0.23** | ECS |
| 80 | 10.7 | 21.4 | P02686 | Myelin basic protein | MBP | 5 | 1.08 | **14.19** | **8.87** |  |
| 84 | 10.2 | 18.3 | *Q5UGI6* | Serine/cysteine proteinase inhibitor clade G member 1 splice variant 2 (Fragment) | SERPING1 | 5 | 1.10 | 0.70 | **0.41** | CCC, EIA, ECS, IIR |
| 115 | 6.4 | 20.0 | P02652 | Apolipoprotein A-II | APOA2 | 2 | 0.61 | **0.48** | 0.65 | LT, EIA, RTW, ECS |
| 15 | 76.0 | 42.2 | *A8K5A4* | cDNA FLJ76826, highly similar to Homo sapiens ceruloplasmin (ferroxidase) (CP), mRNA |  | 63 | **1.74** | **0.13** | 0.94 |  |
| 24 | 49.5 | 33.1 | *B2R950* | cDNA, FLJ94213, highly similar to Homo sapiens pregnancy-zone protein (PZP), mRNA |  | 233 | 0.90 | **0.43** | 1.27 |  |
| 26 | 47.1 | 44.5 | *A8K5J8* | cDNA FLJ75066, highly similar to Homo sapiens complement component 1, r subcomponent (C1R), mRNA |  | 31 | **0.67** | **0.25** | **0.23** |  |
| 41 | 31.4 | 10.2 | *B4E1Z4* | cDNA FLJ55673, highly similar to Complement factor B (EC 3.4.21.47) |  | 13 | **1.41** | **0.41** | **0.44** |  |
| 56 | 18.2 | 26.4 | *Q8WVW5* | Putative uncharacterized protein (Fragment) |  | 9 | **0.09** | 1.71 | 1.16 | FA, RTW |
| 57 | 18.2 | 8.7 | *Q59E99* | Thrombospondin 1 variant (Fragment) |  | 8 | **0.55** | 1.17 | 1.03 | FA, ECS |
| 70 | 13.4 | 16.1 | *B3KS79* | cDNA FLJ35730 fis, clone TESTI2003131, highly similar to ALPHA-1-ANTICHYMOTRYPSIN |  | 7 | 1.01 | 0.51 | **0.38** |  |
| 75 | 12.2 | 9.6 | *B7Z550* | cDNA FLJ59731, highly similar to Complement component C8 beta chain | C8B | 6 | 0.83 | **0.44** | **0.39** |  |
| 145 | 4.6 | 14.6 | *A8K486* | Peptidyl-prolyl cis-trans isomerase***** |  | 2 | 1.96 | **7.05** | **5.45** |  |
| 176 | 3.3 | 2.2 | *Q59GB4* | Dihydropyrimidinase-like 2 variant (Fragment) |  | 1 | 1.38 | **5.40** | 3.84 |  |

***^a^***The list contains quantitative information of the selected proteins from bias and background corrected iTRAQ data set. The denominator is the demographically matched control. Unused and %coverage are parameters related to the confident identification of proteins. This list contains 43 candidates qualified (out of 183) through the initial filters [i.e., unused prot score >3.0 and FDR = 1.1% (confident identification), *p*-value <0.05 (significantly different from 1) for at least one ratio]. **The Significant ratios are indicated in bold**. The uniport accession numbers of the ‘unreviewed’ proteins are indicated in ***italics*** form. The protein whose evidence is available only at the level of transcript is not provided with a gene symbol. The last column provides information about GO or pathway. CCC = complement and coagulation cascade, ECS = extracellular space, EIA = enzyme inhibitor activity, FA = focal adhesion, IIR = innate immune response, LT= lipid transport, RTW = response to wounding. ***Reported as microvesicle or exosome marker by independent studies**.
